# The ischemic time window of ectopic endometrial tissue crucially determines its ability to develop into endometriotic lesions

**DOI:** 10.1038/s41598-022-09577-z

**Published:** 2022-04-04

**Authors:** Jeannette Rudzitis-Auth, Sarah I. Huwer, Claudia Scheuer, Michael D. Menger, Matthias W. Laschke

**Affiliations:** Institute for Clinical and Experimental Surgery, Saarland University, 66421 Homburg/Saar, Germany

**Keywords:** Imaging, Experimental models of disease

## Abstract

Endometriosis develop from shed endometrial fragments via retrograde menstruation. This affects the survival, proliferation and vascularization of the tissue and its final ability to form endometriotic lesions. Within this study, uterine tissue samples from donor mice were precultivated for 24 h or 72 h to simulate avascular periods. Their morphology, microvessel density, apoptotic activity and expression of angiogenesis-related proteins were analyzed in vitro. The formation of endometriotic lesions in vivo was assessed after transplantation of precultivated uterine tissue samples to the abdominal wall and dorsal skinfold chambers by means of high-resolution ultrasound, intravital fluorescence microscopy, histology and immunohistochemistry. In vitro, 72-h-precultivated uterine tissue samples exhibit extensive areas of tissue necrosis and high numbers of apoptotic cells as well as a significantly reduced cell and microvessel density. These samples failed to develop into endometriotic lesions. In contrast, the 24-h-precultivated samples showed, that their early vascularization and growth in vivo was improved when compared to controls. This indicates that avascular periods have a strong impact on the survival of ectopic endometrial tissue and the chance for the development of endometriosis.

## Introduction

Endometriosis is an estrogen-dependent, benign gynecological disease, which affects about 5–10% of women during their reproductive years. It is characterized by the presence of endometriotic lesions, consisting of endometrial stromal and epithelial cells, outside the uterine cavity^1^. These lesions are mainly located on the pelvic organs, i.e. uterus and ovaries, and may also affect the ligaments, deep pelvic nerves and the recto-uterine pouch. Moreover, they can be found on the intestine and the bladder as well as in rare cases in the liver, lung and brain^2^.

Although endometriosis has been known since centuries, the pathogenesis of this common disease still remains unclear. In fact, various theories have been established to explain the development of endometriotic lesions^3^. The coelomic metaplasia theory proposes that the occurrence of endometrial cells in the peritoneal cavity results from the differentiation of mesothelial cells into endometrium-like tissue^4^. Another theory suggests the formation of endometriotic lesions through the activation of embryonic cells of the Mullerian ducts^5^. These processes are thought to be stimulated by hormonal or immunological factors and may also explain the rare presence of endometriotic lesions in males^6^. In a third theory, it is speculated that endometriosis in the pulmonary system, muscles, skin and brain may develop through lymphatic or hematogenous dissemination of endometrial cells^7^.

The most widely quoted endometriosis theory is still Sampson’s implantation theory, postulating the formation of endometriotic lesions by retrogradely shed endometrial tissue via the fallopian tubes into the peritoneal cavity^8^. However, retrograde menstruation is a common physiological event. Accordingly, blood can be found in the peritoneal fluid of 90% of menstruating women^9,10^. Therefore, other important factors must contribute to the onset of endometriosis^11^. These may particularly include pathological alterations in the eutopic endometrium, such as the overproduction of prostaglandins, cytokines, chemokines and matrix metalloproteinases (MMPs) as well as dysregulated proliferation and apoptotic cell signaling pathways^12,13^. Besides, an impaired immune system with defect natural killer cells and a reduced T-cell cytotoxicity may lead to a reduced clearance of ectopic endometrial tissue inside the peritoneal cavity^14^. Finally, the tissue integrity of refluxed endometrium seems to be essential for the establishment of endometriotic lesions^15^. In fact, experimental studies indicate that an intact architecture of freshly isolated endometrial fragments with stromal tissue and endometrial glands is a crucial prerequisite for the formation of endometriotic lesions^15,16^. However, it should be considered that in women the passage of such shed endometrial fragments from the uterus into the peritoneal cavity and their final engraftment may require several hours to days. During this period, the endometrial tissue lacks a blood supply and therefore may at least partly suffer from hypoxia. It is unknown how this ischemic time window affects the survival, proliferation, angiogenesis and inflammatory activity of the tissue and, hence, its final ability to develop into endometriotic lesions.

To shed more light on this question, we herein precultivated uterine tissue samples from donor mice for 24 h or 72 h to simulate in vitro a variable period of retrograde menstruation, while freshly isolated tissue samples served as control. Subsequently, we analyzed their morphology, microvessel density, viability, apoptotic activity, tissue hypoxia and expression of angiogenesis-related proteins. In addition, freshly isolated and precultivated uterine tissue samples were transplanted into the abdominal cavity and the dorsal skinfold chamber of recipient mice to investigate the formation of endometriotic lesions as well as their vascularization, proliferation and immune cell infiltration by means of high-resolution ultrasound, intravital fluorescence microscopy, histology and immunohistochemistry.

## Results

### Histomorphological characterization of precultivated uterine tissue samples

**Figure 1:**
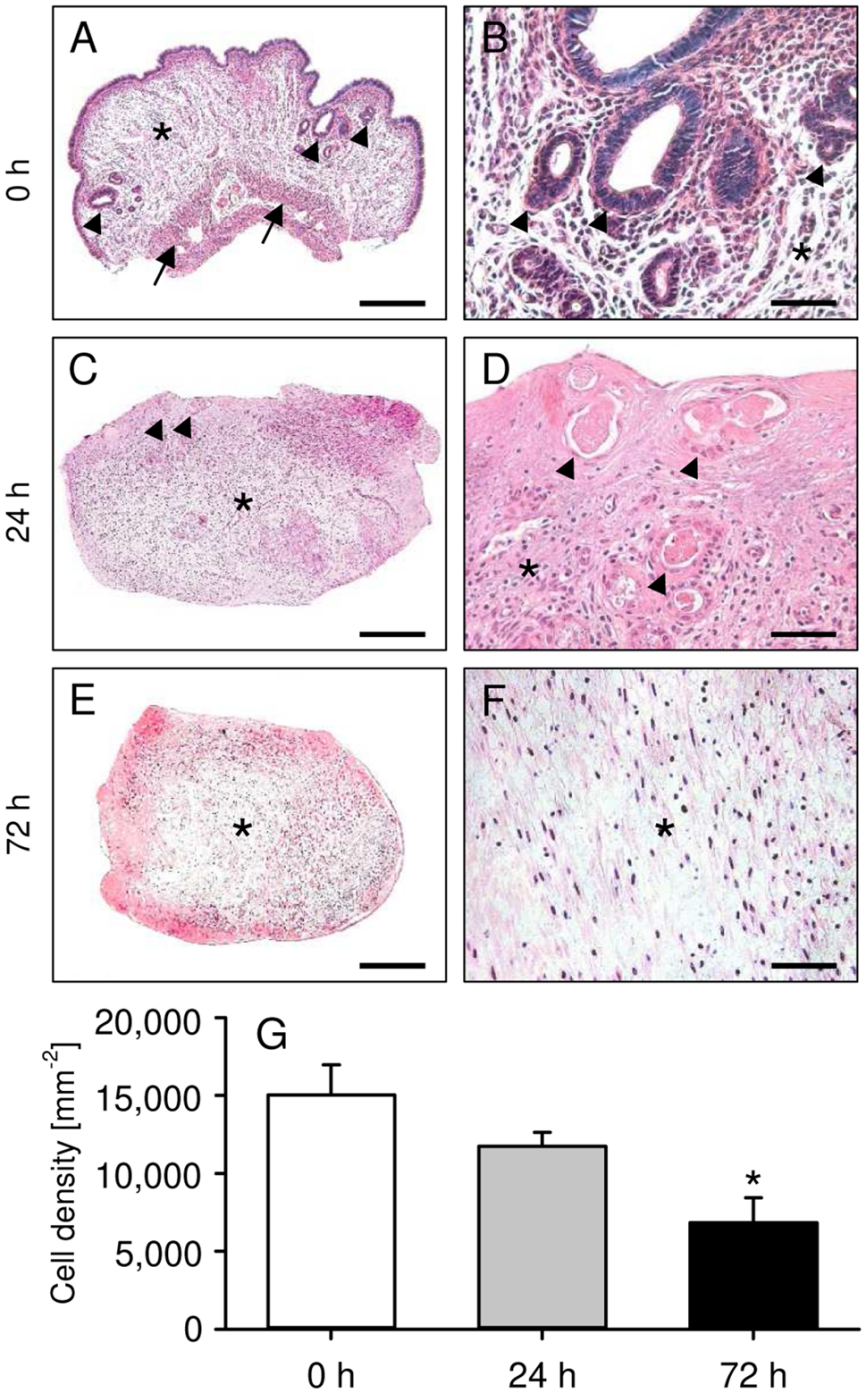
(**A**–**F**) HE-stained sections of freshly isolated (0 h; **A**,**B**) as well as 24-h- (**C**,**D**) and 72-h-precultivated (**E**,**F**) uterine tissue samples from C57BL/6-TgN(ACTB-EGFP)1Osb/J donor mice (arrows = myometrium with perimetrium; arrowheads = endometrial glands; asterisks = endometrial stroma). Scale bars: (**A**,**C**,**E**) 300 µm; (**B**,**D**,**F**) 60 µm. (**G**) Cell density (mm^−2^) in freshly isolated (0 h; white bars; n = 5) as well as 24-h- (grey bars; n = 5) and 72-h-precultivated (black bars; n = 5) uterine tissue samples from C57BL/6-TgN(ACTB-EGFP)1Osb/J donor mice. Mean ± SEM; *P < 0.05 vs. 0 h.

**Figure 2:**
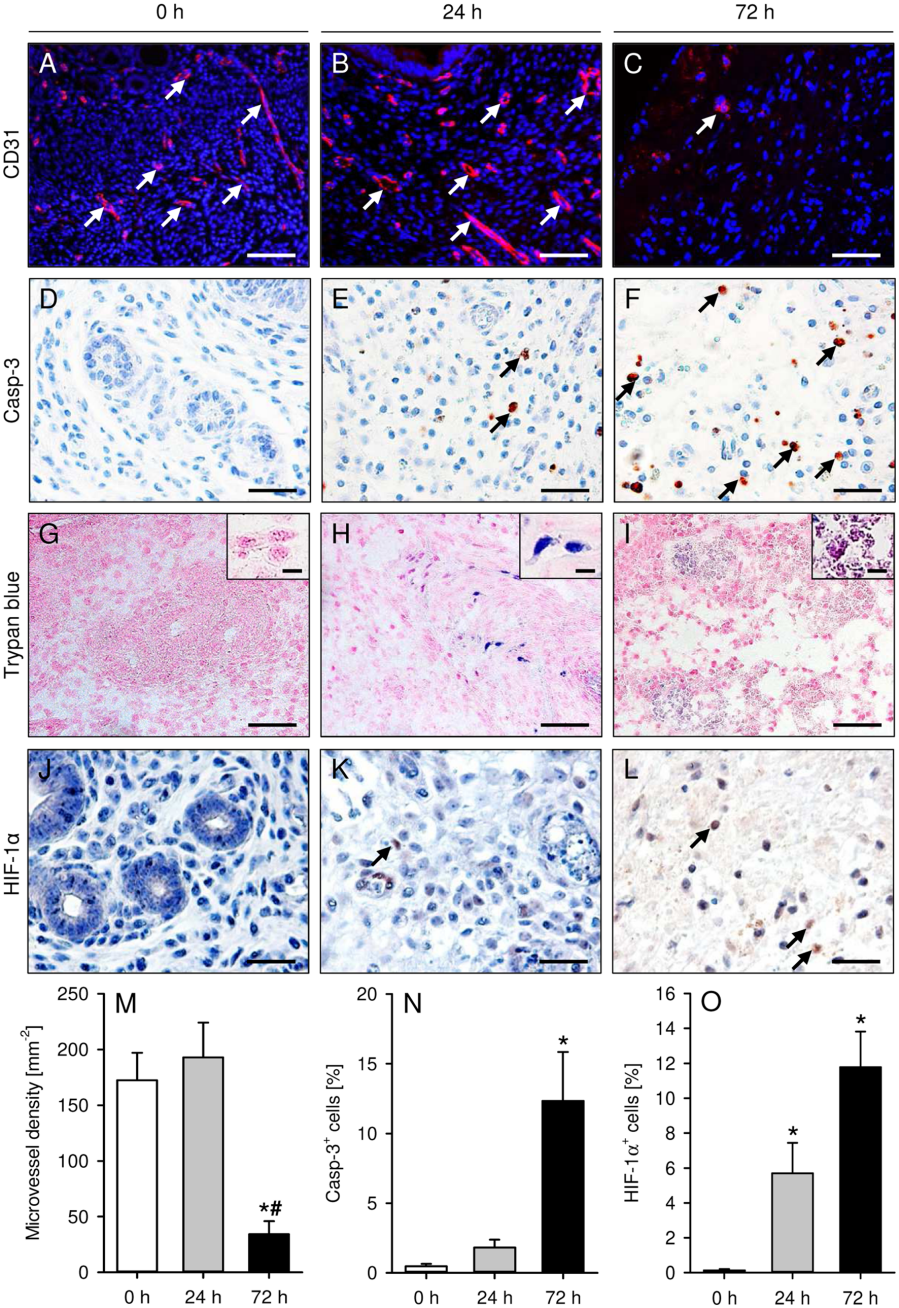
(**A**–**F**) Immunofluorescent and immunohistochemical detection of microvessels (**A**–**C**, arrows) and Casp-3^+^ apoptotic cells (**E**,**F**, arrows) in freshly isolated (0 h; **A**,**D**) as well as 24-h- (**B**,**E**) and 72-h-precultivated (**C**,**F**) uterine tissue samples from C57BL/6-TgN(ACTB-EGFP)1Osb/J donor mice. The immunofluorescent sections (**A**–**C**) were stained with Hoechst 33342 to identify cell nuclei (blue) and an antibody against CD31 for the detection of microvessels (red). To detect apoptotic cells (**D**–**F**), sections were stained with an antibody against Casp-3. Scale bars: (**A**–**C**) = 60 µm; (**D**–**F**) = 30 µm. (**G**–**I**) Histological detection of trypan blue^+^ dead cells in freshly isolated (0 h; **G**) as well as 24-h- (**H**) and 72-h-precultivated (**I**) uterine tissue samples from C57BL/6-TgN(ACTB-EGFP)1Osb/J donor mice. Inserts in (**G**–**I**) show the morphology of individual cells. Scale bars: (**G**–**I**) = 50 µm; inserts = 7 µm. (**J**–**L**) Immunohistochemical detection of HIF-1α^+^ hypoxic cells (**K**,**L**, arrows) in freshly isolated (0 h; **J**) as well as 24-h- (**K**) and 72-h-precultivated (**L**) uterine tissue samples from C57BL/6-TgN(ACTB-EGFP)1Osb/J donor mice. To detect hypoxic cells, sections were stained with an antibody against HIF-1α. Scale bars: 30 µm. (**M**–**O**) Microvessel density (**M**, mm^−2^), Casp-3^+^ apoptotic cells (**N**, %) and HIF-1α^+^ hypoxic cells (**O**, %) in freshly isolated (0 h; white bars; n = 5–9) as well as 24-h- (grey bars; n = 5–9) and 72-h-precultivated (black bars; n = 5–8) uterine tissue samples from C57BL/6-TgN(ACTB-EGFP)1Osb/J donor mice. Mean ± SEM; *P < 0.05 vs. 0 h; ^#^P < 0.05 vs. 24 h.

In a first set of experiments, we analyzed the morphology of freshly isolated as well as 24-h- and 72-h-precultivated uterine tissue samples by means of histology and immunohistochemistry (Figs. 1 and 2). Freshly isolated uterine tissue samples exhibited a physiological tissue architecture, consisting of endometrium, myometrium and perimetrium (Fig. 1A,B). After 24 h of precultivation, this layered structure was no longer clearly visible (Fig. 1C). Moreover, most of the endometrial glands had regressed (Fig. 1D). The morphology of 72-h-precultivated uterine tissue samples was massively altered with extensive areas of tissue necrosis and, consequently, a significantly reduced cell density when compared to freshly isolated controls (Fig. 1E–G).

Of interest, freshly isolated and 24-h-precultivated uterine tissue samples exhibited a comparable microvessel density (Fig. 2A,B,M), indicating that the microvasculature of the isolated endometrial tissue survived the ischemic period of 24 h. In contrast, the microvessel density of 72-h-precultivated uterine tissue samples was significantly lower (Fig. 2C,M). In line with these findings, we also detected a markedly higher number of cleaved caspase (Casp)-3^+^ apoptotic cells within 72-h-precultivated uterine tissue samples when compared to the other two groups (Fig. 2D–F,N). Moreover, trypan blue stainings revealed that freshly isolated uterine tissue samples did not contain any trypan blue^+^ dead cells (Fig. 2G). In 24-h-precultivated samples we detected only a very few dead cells (˂ 1%) (Fig. 2H), indicating a preserved viability. In contrast, 72-h-precultivated uterine tissue samples were full of cell debris, which could not reliably be dyed anymore with trypan blue (Fig. 2I).

Finally, additional sections were stained with an antibody against hypoxia-inducible factor (HIF)-1α. This demonstrated that 24-h- and 72-h-precultivated uterine tissue samples contained a significantly higher fraction of hypoxic cells when compared to freshly isolated controls (Fig. 2J–L,O).

### Protein expression of precultivated uterine tissue samples

We next assesed the expression of 53 angiogenesis-related proteins in freshly isolated as well as 24-h- and 72-h-precultivated pooled uterine tissue samples by means of a proteome profiler mouse angiogenesis array. Of note, we detected an unusually high expression of distinct proteins in the group of 72-h-precultivated uterine tissue samples, although our analyses on cell viability demonstrated that these samples mainly contained dying and non-viable cells or even cellular debris (Supplementary Table S1). Because non-specific binding of antibodies is a well-known problem in the analysis of such samples^17,18^, we therefore excluded the expression data of this group to prevent misleading interpretations due to false-positive artefacts. Precultivation for 24 h upregulated the expression of most of the analyzed proteins when compared to freshly isolated controls (Table 1). The highest upregulation was found for the pro-angiogenic factors Cyr61 and keratinocyte chemoattractant (KC) (Table 1).

**Table 1:** Expression of angiogenesis-related proteins in 24-h-precultivated and freshly isolated uterine tissue samples as assessed by a proteome profiler mouse angiogenesis array.

Protein	Mean pixel density (mean ± SEM)		Fold change (%)
	0 h	24 h	24 h
Cyr61/CCN1, IGFBP-10	3279 ± 19	71,506 ± 1501	2181
KC/CXCL1/CINC-1/GRO-alpha	489 ± 208	8661 ± 252	1771
ADAMTS1/METH1	3872 ± 137	48,197 ± 1158	1245
Thrombospondin-2/TSP-2	8025 ± 82	80,818 ± 1017	1007
MCP-1/CCL2/JE	4931 ± 250	44,474 ± 226	902
DLL4	1566 ± 43	13,217 ± 1011	844
DPP IV/CD26	7471 ± 53	45,106 ± 734	604
PIGF-2	4962 ± 376	27,994 ± 1027	564
Amphiregulin/AR	1180 ± 171	6566 ± 12	556
MMP-3	21,410 ± 30	112,140 ± 780	524
Serpin E1/PAI-1	27,886 ± 521	138,533 ± 6104	497
IL-1alpha	4489 ± 202	21,424 ± 139	477
Osteopontin/OPN	28,734 ± 186	135,821 ± 762	473
KGF/FGF-7	823 ± 75	3852 ± 62	468
Endoglin/CD105	5148 ± 675	19,102 ± 1203	371
GM-CSF	587 ± 112	1955 ± 34	333
Fractalkine/CX3CL 1	3458 ± 471	10,474 ± 2011	303
HB-EGF	2220 ± 10	6621 ± 277	298
TIMP-1	4024 ± 343	11,882 ± 296	295
PDGF-AA	2299 ± 144	6292 ± 321	274
IP-10/CXCL 10	2303 ± 253	6042 ± 131	262
Angiopoietin-1/Ang-1	1950 ± 209	4937 ± 123	253
IGFBP-3	59,053 ± 599	133,221 ± 3273	226
Pentraxin-3/PTX3/TSG-14	4486 ± 194	9420 ± 680	210
Endothelin-1/ET-1	8289 ± 11	16,646 ± 687	201
PDGF-AB/BB	3455 ± 105	6801 ± 91	197
Platelet Factor 4/CXCL4/PF4	23,151 ± 291	44,910 ± 822	194
VEGF/VPF	1146 ± 151	2178 ± 304	190
FGF acid/FGF-1/ECGF/HBGF-1	26,308 ± 25	46,657 ± 309	177
MIP-1alpha	3095 ± 109	5214 ± 21	169
IL-10/CSIF	2479 ± 410	3880 ± 20	157
Angiopoietin-3/Ang-3	2765 ± 308	4316 ± 231	156
VEGF B/VRF	4444 ± 44	6742 ± 211	152
Proliferin	3814 ± 230	5725 ± 585	150
Endostatin/Collagen VIII	41,865 ± 1410	62,267 ± 248	149
HGF	25,854 ± 425	37,891 ± 2033	147
CXCL 16	17,205 ± 343	23,265 ± 1348	135
PD-ECGF	1932 ± 24	2392 ± 519	124
Angiogenin/ANG	5489 ± 326	6639 ± 247	121
Leptin/OB	2983 ± 506	3567 ± 93	120
Prolactin/PRL	2609 ± 199	3007 ± 319	115
FGF basic/FGF-2	13,601 ± 448	15,613 ± 476	115
IL-1beta	520 ± 77	582 ± 266	112
IGFBP-2	7215 ± 142	8070 ± 797	112
TIMP-4	2262 ± 308	2389 ± 297	106
IGFBP-1	3551 ± 65	3515 ± 10	99
NOV/CCN3/IGFBP-9	31,010 ± 1903	30,571 ± 2902	99
MMP-9	112,331 ± 21	107,283 ± 2339	96
Coagulator Factor III/Tissue Factor/TF	54,603 ± 79	52,105 ± 2272	95
MMP-8	7085 ± 418	6349 ± 448	90
SDF-1/CXCL 12	34,391 ± 1770	30,537 ± 2293	89
EGF	955 ± 28	844 ± 129	88
Serpin F1/PEDF	11,456 ± 43	9101 ± 613	79

Data are presented as mean pixel density ± SEM of two technical replicates and as fold change in % of freshly isolated controls.

### Development of endometriotic lesions

**Figure 3:**
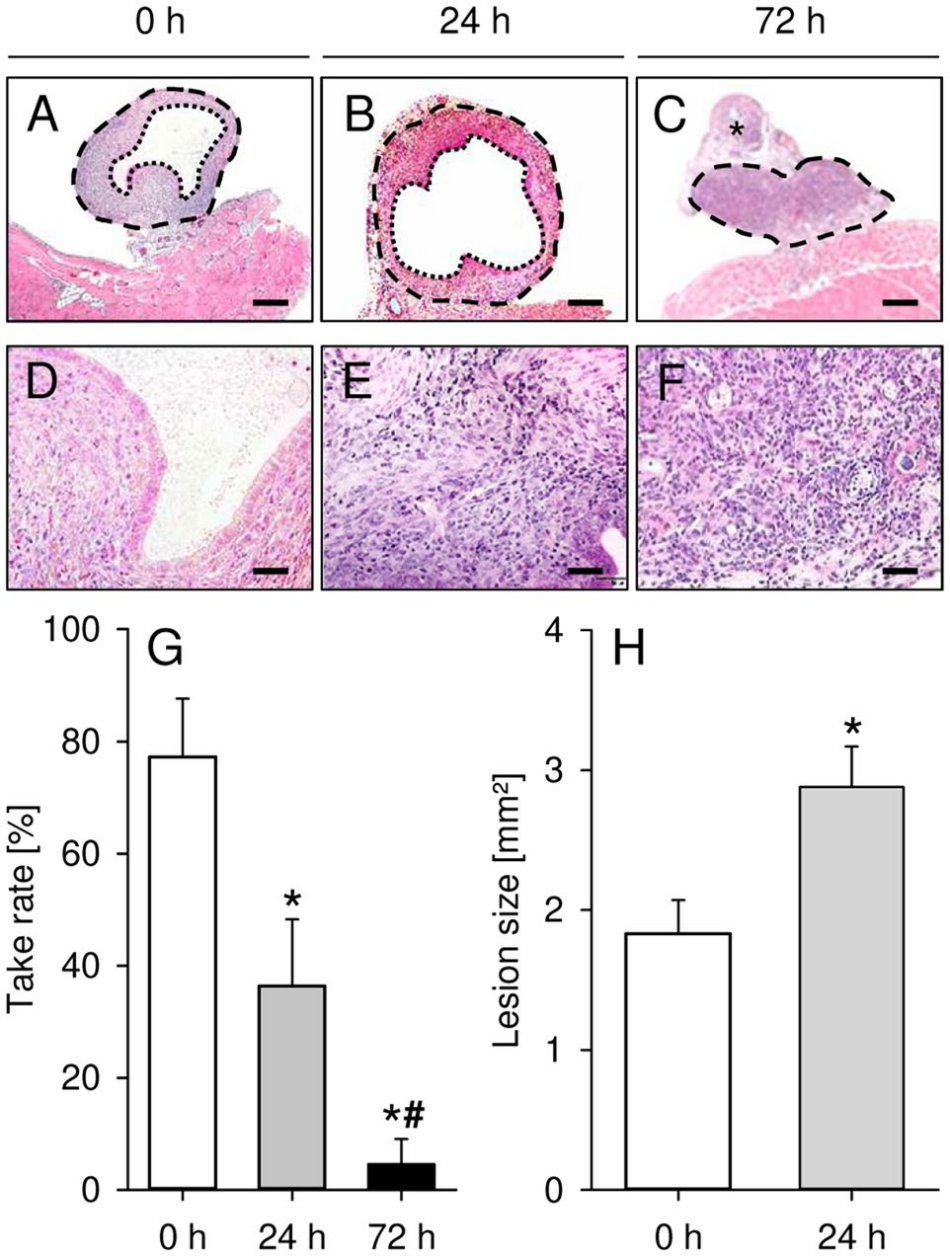
(**A**–**F**) HE-stained sections of endometriotic lesions (broken lines = borders; dotted lines = cyst-like dilated endometrial glands; asterisk = suture) on day 28 after surgical induction by fixation of freshly isolated (0 h; **A**,**D**) as well as 24-h- (**B**,**E**) and 72-h-precultivated (**C**,**F**) uterine tissue samples from C57BL/6-TgN(ACTB-EGFP)1Osb/J donor mice to the peritoneal wall of C57BL/6J recipient mice. Scale bars: (**A**–**C**) = 200 µm; (**D**–**F**) = 35 µm. (**G**,**H**) Take rate (**G**, %) and lesion size (**H**, mm^2^) of endometriotic lesions on day 28 after transplantation of freshly isolated (0 h; white bars; n = 10–11) as well as 24-h- (grey bars; n = 6–11) and 72-h-precultivated (black bars; n = 11) uterine tissue samples from C57BL/6-TgN (ACTB-EGFP)1Osb/J donor mice to the peritoneal wall of C57BL/6J recipient mice. Mean ± SEM; *P < 0.05 vs. 0 h; ^#^P < 0.05 vs. 24 h.

In a second set of experiments, we induced endometriotic lesions by suturing freshly isolated and precultivated uterine tissue samples from C57BL/6-TgN(ACTB-EGFP)1Osb/J donor mice to the abominal wall of C57BL/6J recipient mice. After 28 days, the grafts were analyzed by histology to determine the take rate (Fig. 3A–G). This analysis revealed that the take rate progressively decreased with the duration of precultivation. While ~ 80% of freshly isolated uterine tissue samples developed into endometriotic lesions consisting of endometrial glands and stroma, the take rate of 24-h-precultivated samples was only ~ 40% (Fig. 3G). However, additional caliper measurements revealed that the overall size of the lesions orginating from 24-h-precultivated tissue samples was significantly higher when compared to freshly isolated controls (Fig. 3H). Moreover, we found that only one out of 22 72-h-precultivated uterine tissue samples developed into a typical lesion, which corresponds to a take rate of only ~ 5% (Fig. 3G). Hence, we excluded this group from all further quantitative analyses in the peritoneal model.

**Figure 4:**
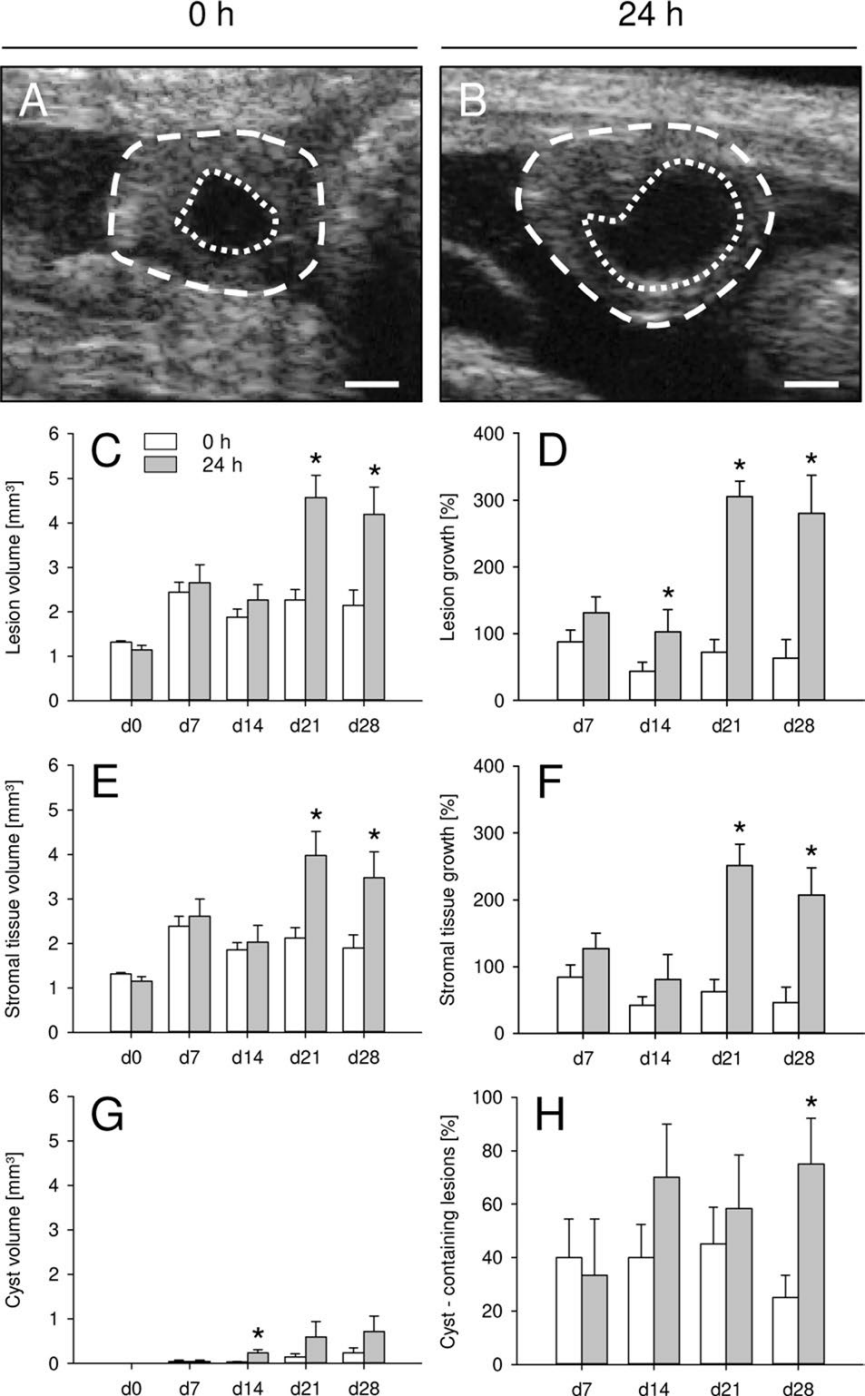
(**A**,**B**) High-resolution ultrasound imaging of endometriotic lesions (broken lines = borders, dotted lines = cyst-like dilated endometrial glands) on day 28 after transplantation of freshly isolated (0 h; **A**) and 24-h-precultivated (**B**) uterine tissue samples from C57BL/6-TgN(ACTB-EGFP)1Osb/J donor mice to the peritoneal wall of C57BL/6J recipient mice. Scale bars: 500 µm. (**C**–**H**) Overall lesion volume (**C**, mm^3^), lesion growth (**D**, %), stromal tissue volume (**E**, mm^3^), stromal tissue growth (**F**, %), cyst volume (**G**, mm^3^) and cyst-containing lesions (**H**, %) of endometriotic lesions directly after transplantation (d0) as well as on days 7, 14, 21 and 28 after transplantation of freshly isolated (0 h; white bars; n = 10) and 24-h-precultivated (grey bars; n = 6) uterine tissue samples from C57BL/6-TgN (ACTB-EGFP)1Osb/J donor mice to the peritoneal wall of C57BL/6J recipient mice. Mean ± SEM; *P < 0.05 vs. 0 h.

The growth and cyst formation of newly developing endometriotic lesions was repeatedly analyzed by means of high-resolution ultrasound imaging throughout the 28-days in vivo experiment (Fig. 4A,B). Directly after transplantation into the peritoneal cavity, freshly isolated and 24-h-precultivated uterine tissue samples exhibited a comparable initial volume, which increased over time (Fig. 4C). However, the growth rate of lesions in the group of 24-h-precultivated tissue samples was significantly higher between day 14 and 28 when compared to controls (Fig. 4D). Accordingly, these lesions also exhibited a markedly higher overall lesion volume, stromal tissue volume and stromal tissue growth on day 21 and 28 (Fig. 4C,E,F). The cyst volumes of the lesions in both groups remained rather low during the entire observation period (Fig. 4G). However, on day 28 the number of detectable cyst-like dilated endometrial glands was significantly higher in lesions originating from 24-h-precultivated uterine tissue samples when compared to controls (Fig. 4H).

### Vascularization, proliferation and apoptosis of endometriotic lesions

**Figure 5:**
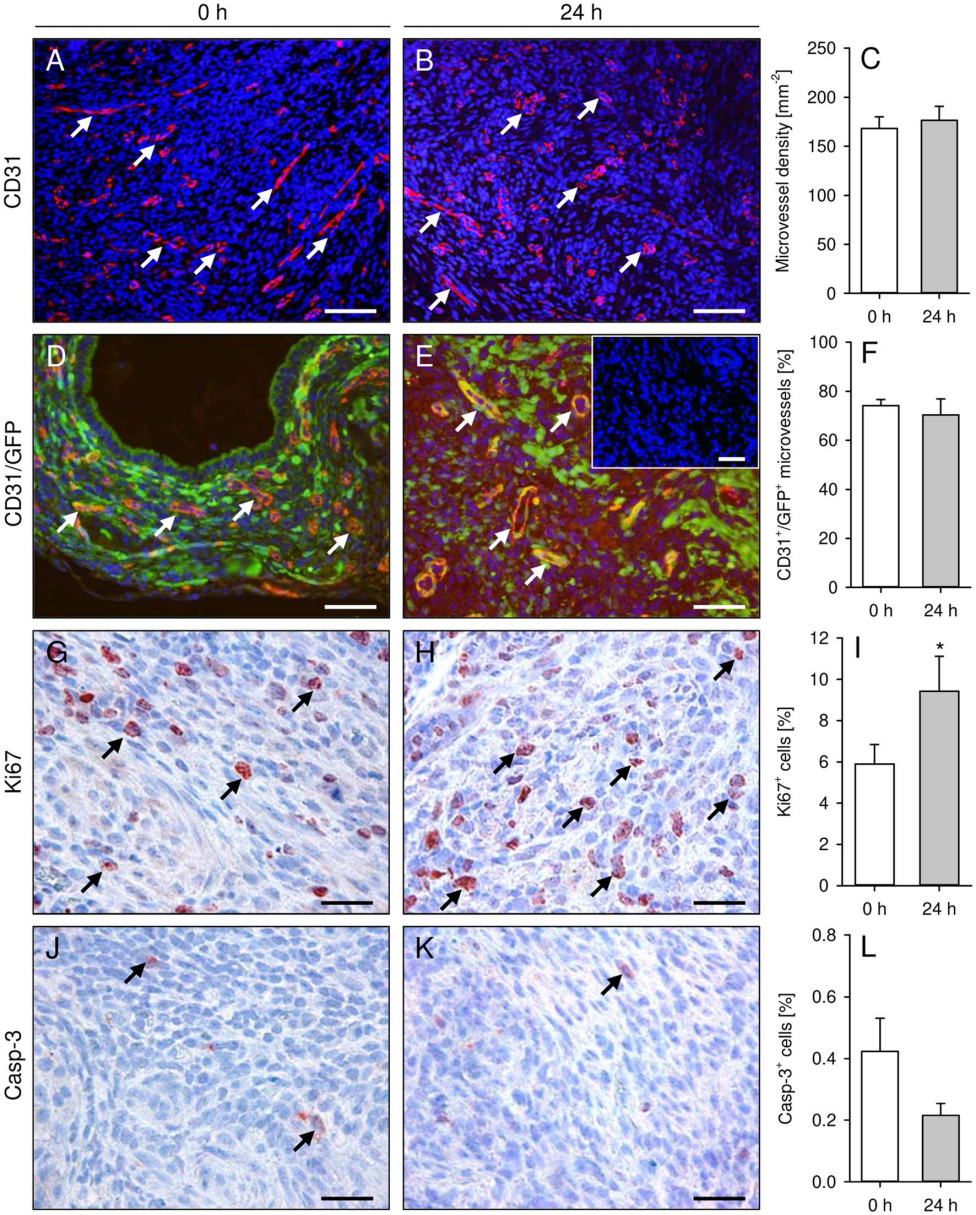
(**A**,**B**,**D**,**E**) Immunofluorescent detection of CD31^+^ microvessels (**A**,**B**; arrows) and CD31^+^/GFP^+^ microvessels (**D**,**E**; arrows) in endometriotic lesions on day 28 after transplantation of freshly isolated (0 h; **A**,**D**) and 24-h-precultivated (**B**,**E**) uterine tissue samples from C57BL/6-TgN(ACTB-EGFP)1Osb/J donor mice to the peritoneal wall of C57BL/6J recipient mice. The immunofluorescent sections were stained with Hoechst 33342 to identify cell nuclei (blue), an antibody against CD31 for the detection of microvessels (red) and an antibody against GFP to detect GFP^+^ microvessels (green; **D**,**E**). Insert in E shows negative staining control. Scale bars: (**A**,**B**) = 60 µm; (**D**,**E**) insert = 40 µm. (**G**,**H**,**J**,**K**) Immunohistochemical detection of Ki67^+^ proliferating cells (**G**,**H**; arrows) and Casp-3^+^ apoptotic cells (**J**,**K**; arrows) in endometriotic lesions on day 28 after transplantation of freshly isolated (0 h; **G**,**J**) and 24-h-precultivated (**H**,**K**) uterine tissue samples from C57BL/6-TgN(ACTB-EGFP)1Osb/J donor mice to the peritoneal wall of C57BL/6J recipient mice. To detect proliferating (**G**,**H**) and apoptotic cells (**J**,**K**), the sections were stained with an antibody against Ki67 and Casp-3. Scale bars: (**G**,**H**,**J**,**K**) = 25 µm. (**C**,**F**,**I**,**L**): Microvessel density (**C**, mm^−2^), CD31^+^/GFP^+^ microvessels (**F**, %), Ki67^+^ proliferating cells (**I**, %) and Casp-3^+^ apoptotic cells (**L**, %) in endometriotic lesions on day 28 after transplantation of freshly isolated (0 h; white bars; n = 10) and 24-h-precultivated (grey bars; n = 6) uterine tissue samples from C57BL/6-TgN(ACTB-EGFP)1Osb/J donor mice to the peritoneal wall of C57BL/6J recipient mice. Mean ± SEM; *P < 0.05 vs. 0 h.

At the end of the in vivo experiments, the endometriotic lesions were additionally analyzed by immunohistochemistry. The lesions developing from freshly isolated and 24-h-precultivated uterine tissue samples exhibited a comparable density of CD31^+^ microvessels (Fig. 5A–C). Furthermore, the fraction of CD31^+^/green fluorescent protein (GFP)^+^ microvessels was comparably high (~ 70–75%) in both groups (Fig. 5D–F). These findings indicate that the major part of the lesions’ final microvasculature originated from those GFP^+^ microvessels, which were originally present in the uterine tissue samples of the C57BL/6-TgN(ACTB-EGFP)1Osb/J donor mice. In contrast, only a few GFP^-^ microvessels progressively grew from the surrounding host tissue into the lesions and developed interconnections to the pre-existing GFP^+^ microvessels during the 28-day observation period.

In line with the results of our ultrasound imaging analyses, we additionally detected a significantly higher number of proliferating Ki67^+^ stromal cells in lesions originating from 24-h-precultivated uterine tissue samples when compared to controls (Fig. 5G–I). Moreover, we found a comparably low fraction of Casp-3^+^ apoptotic stromal cells within the lesions of the two groups with a tendency towards a lower level in the lesions originating from 24-h-precultivated uterine tissue samples (Fig. 5J–L).

### Immune cell infiltration into endometriotic lesions

**Figure 6:**
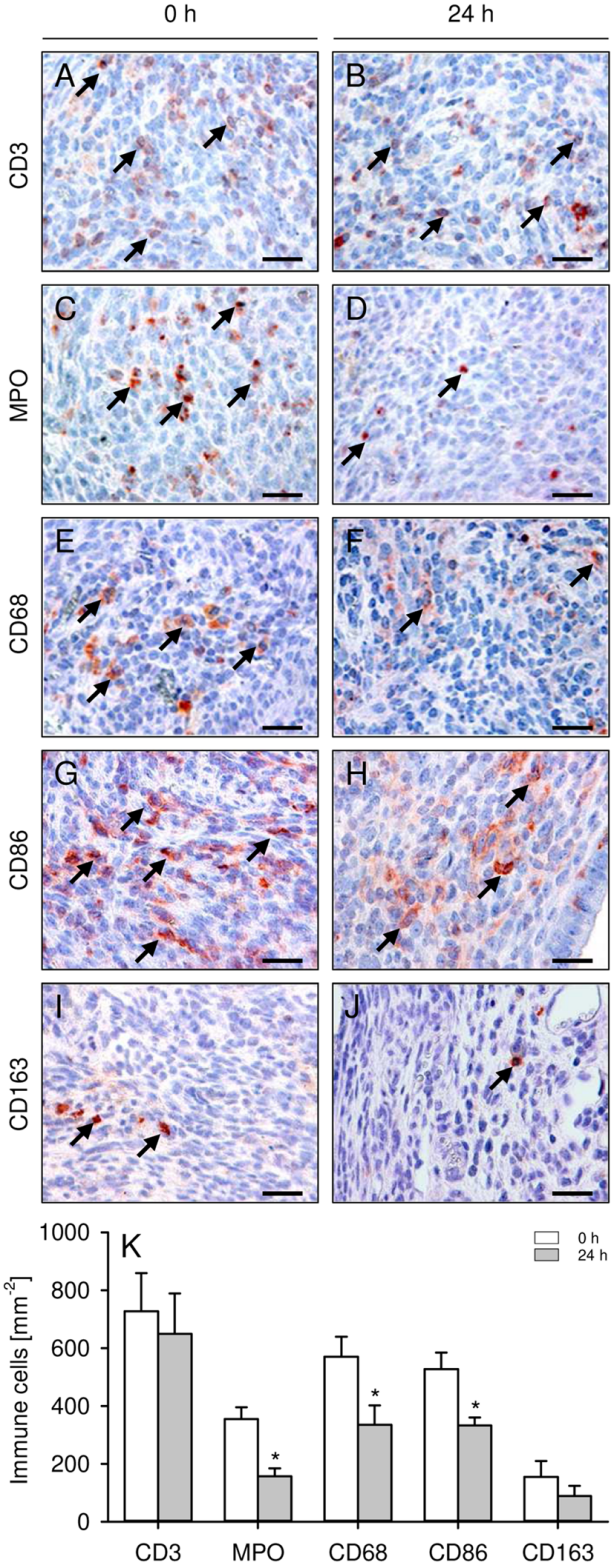
(**A**–**J**) Immunohistochemical detection of CD3^+^ lymphocytes (**A**,**B**; arrows), MPO^+^ neutrophilic granulocytes (**C**,**D**; arrows), CD68^+^ macrophages (**E**,**F**; arrows), CD86^+^ M1 macrophages (**G**,**H**; arrows) and CD163^+^ M2 macrophages (**I**,**J**; arrows) in endometriotic lesions on day 28 after transplantation of freshly isolated (0 h; **A**,**C**,**E**,**G**,**I**) and 24-h-precultivated (**B**,**D**,**F**,**H**,**J**) uterine tissue samples from C57BL/6-TgN(ACTB-EGFP)1Osb/J donor mice to the peritoneal wall of C57BL/6J recipient mice. Scale bars: 25 µm. (**K**) CD3^+^ lymphocytes (mm^−2^), MPO^+^ neutrophilic granulocytes (mm^−2^), CD68^+^ macrophages (mm^−2^), CD86^+^ M1 macrophages (mm^−2^) and CD163^+^ M2 macrophages (mm^−2^) in endometriotic lesions on day 28 after transplantation of freshly isolated (0 h; white bars; n = 10) and 24-h-precultivated (grey bars; n = 6) uterine tissue samples from C57BL/6-TgN(ACTB-EGFP)1Osb/J donor mice to the peritoneal wall of C57BL/6J recipient mice. Mean ± SEM; *P < 0.05 vs. 0 h.

Because inflammatory processes are involved in the pathogenesis of endometriosis^19^, we further investigated the immune cell infiltration into endometriotic lesions. Immunohistochemical stainings of different immune cell subpopulations revealed an infiltration of the lesions by CD3^+^ lymphocytes, CD68^+^ macrophages and MPO^+^ neutrophilic granulocytes (Fig. 6A–F). Notably, the lesions originating from 24-h-precultivated uterine tissue samples contained significantly less MPO^+^ neutrophilic granulocytes and CD68^+^ macrophages when compared to controls (Fig. 6K). More detailed analyses revealed that the number of CD86^+^ M1 macrophages was significantly reduced within endometriotic lesions originating from 24-h-precultivated uterine tissue samples when compared to controls, whereas the number of CD163^+^ M2 macrophages did not differ between the two groups (Fig. 6G–K).

### Early vascularization of endometriotic lesions in dorsal skinfold chambers

**Figure 7:**
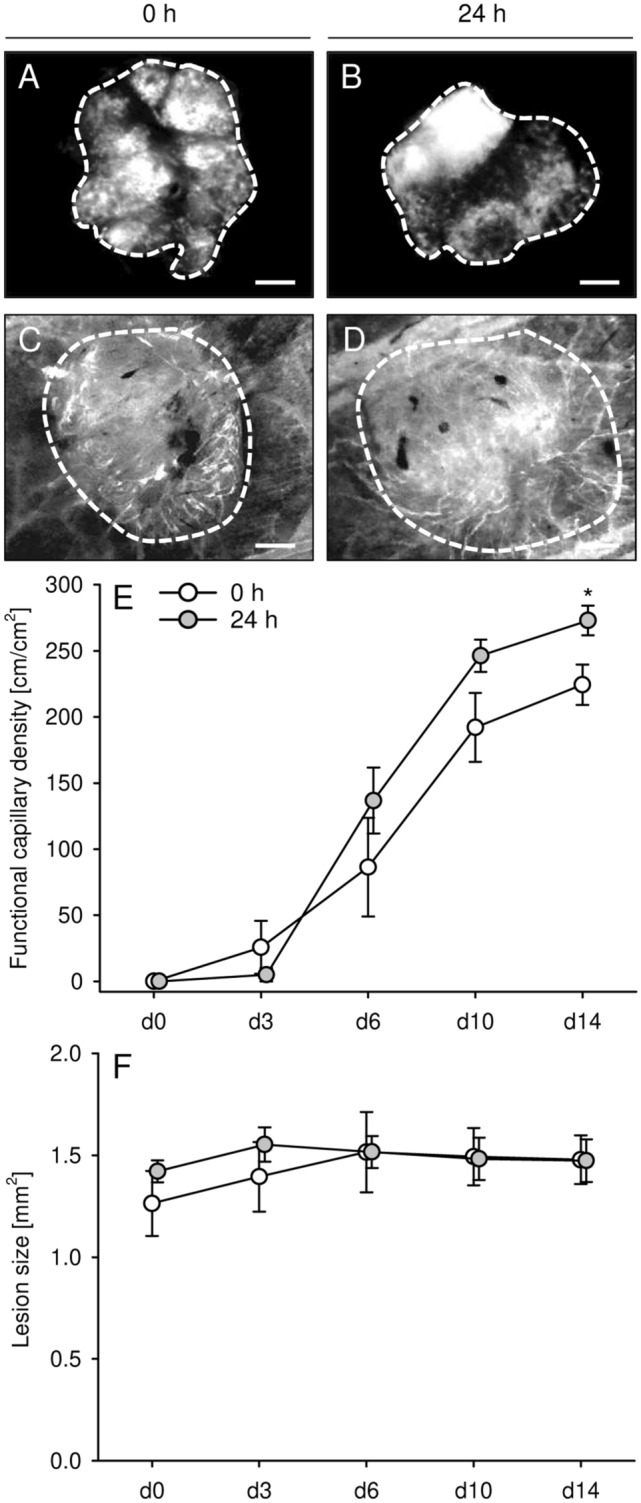
(**A**–**D**) Intravital fluorescent microscopic images of endometriotic lesions (broken lines = borders) on day 0 (**A**,**B**) and day 14 (**C**,**D**) after transplantation of freshly isolated (0 h; **A**,**C**) and 24-h-precultivated (**B**,**D**) endometrial fragments from C57BL/6-TgN(ACTB-EGFP)1Osb/J donor mice into the dorsal skinfold chamber of C57BL/6J recipient mice. Microscopy was performed in blue light epi-illumination without (**A**,**B**) or with 5% FITC-labeled dextran for contrast enhancement by staining of plasma (**C**,**D**). Scale bars: 270 µm. (**E**,**F**) Functional capillary density (**E**, cm/cm^2^) and size (**F**, mm^2^) of endometriotic lesions directly after transplantation (d0) as well as on days 3, 6, 10 and 14 day after transplantation of freshly isolated (0 h; white bars; n = 5) and 24-h-precultivated (grey bars; n = 7) endometrial fragments from C57BL/6-TgN(ACTB-EGFP)1Osb/J donor mice into dorsal skinfold chambers of C57BL/6J recipient mice. Mean ± SEM. *P < 0.05 vs. 0 h.

In a final set of experiments, we transplanted freshly isolated as well as 24-h- and 72-h-precultivated endometrial fragments from C57BL/6-TgN(ACTB-EGFP)1Osb/J donor mice into the dorsal skinfold chambers of C57BL/6J recipient animals to study their early vascularization. Due to their GFP signal, the fragments could be easily detected within the GFP^-^ host tissue by means of intravital fluorescence microscopy (Fig. 7A,B). As performed in the peritoneal endometriosis model, we determined the take rate of the grafts by histology. This analysis revealed that ~ 60% of freshly isolated and ~ 80% of 24-h-precultivated endometrial fragments developed into endometriotic lesions after transplantation. In contrast, only ~ 13% 72-h-precultivated grafts finally exhibited the typical morphology of endometriotic lesions with stromal and glandular cells. Hence, this group was excluded from further quantitative analyses.

Repetitive intravital fluorescence microscopy showed the progressive formation of blood-perfused microvascular networks within both freshly isolated and 24-h-precultivated endometrial grafts throughout the 14-day observation period (Fig. 7C–E). However, at the end of the experiments the functional capillary density of 24-h-precultivated endometrial fragments was significantly higher when compared to freshly isolated controls (Fig. 7E). There were no significant differences in lesion sizes between the two groups (Fig. 7F).

## Discussion

Retrograde menstruation represents a key process in the pathogenesis of endometriosis^20^. During the passage from the uterus into the peritoneal cavity shed endometrial fragments lack a blood supply and, thus, suffer from hypoxic stress. This may markedly affect their viability, tissue integrity as well as their angiogenic, inflammatory and proliferative activity. Our novel findings now indicate that this ischemic time window crucially determines the ability of ectopic endometrial tissue to develop into endometriotic lesions.

To simulate in vitro different periods, in which the tissue lacks a blood supply, we herein precultivated uterine tissue samples from donor mice at 37 °C for 24 h or 72 h in standard cell culture medium in a humidified atmosphere and 5% CO_2_. We are aware that this experimental setting does not exactly reflect the conditions to which endometrial fragments are exposed to during retrograde menstruation through the fallopian tubes and their subsequent distribution inside the peritoneal cavity. In fact, the physiological oxygen concentration in the female reproductive tract and in the peritoneal cavity is much lower than under atmospheric conditions^21,22^. Hence, in vivo endometrial fragments may suffer more from hypoxia when compared to the fragments in our in vitro setting. On the other hand, it is well known that cells grown in a standard normoxic oxygen concentration may actually be exposed to hypoxia to near-anoxic conditions at the cellular level dependent on the density of the cell layer or the depth of the overlying medium^23^. In line with this finding, we herein detected significantly higher fractions of HIF-1α^+^ cells within 24-h- and 72-h-precultivated uterine tissue samples when compared to freshly isolated, non-cultivated controls. In addition, it should be considered that the extent of oxygen diffusion from surrounding tissues may markedly vary between individual endometrial fragments and over time dependent on their in situ localization. Moreover, they may exhibit different shapes and sizes, which crucially determine their survival by oxygen diffusion. Furthermore, the time that is required for the successful engraftment of shed endometrial fragments inside the peritoneal cavity of endometriosis patients is completely unknown. Therefore, our approach should not be interpreted as an attempt to exactly mimic the in vivo situation during retrograde menstruation, but to provide a standardized experimental setting for our proof-of-principle study. For these reasons, it could be of interest to further confirm our results in future studies by means of alternative models of tissue ischemia. For this purpose, endometrial ischemia may be induced in vivo by uterine artery occlusion or by placing freshly excised uterine tissue samples in the peritoneum for 24 and 72 h prior to suturing on the peritoneal wall.

In a first set of experiments, we could demonstrate that the physiological morphology of uterine tissue samples is progressively lost during precultivation. However, we still detected endometrial glands within 24-h-precultivated samples. Moreover, the cell density, viability, microvessel density and fraction of apoptotic cells within these samples did not markedly differ from that of freshly isolated controls. This is an important finding considering the fact that an intact tissue integrity has been reported to be essential for the establishment of endometriotic lesions in different endometriosis models^15,24^. In contrast, 72-h-precultivated uterine tissue samples exhibited extensive areas of tissue necrosis, cellular debris and high numbers of apoptotic cells as well as a significantly reduced cell and microvessel density. Accordingly, these samples also failed to develop into endometriotic lesions after in vivo implantation. This indicates that the onset of endometriosis is only possible in a critical time window during which shed endometrial tissue must successfully reach the peritoneal cavity and engraft at ectopic sites. It may be speculated that pathological changes of the uterus function, such as the known hyper- and dysperistalsis in endometriosis patients^25,26^, provide ideal conditions to fulfil this requirement.

In our mouse model of surgically induced endometriosis we found a significantly lower take rate of 24-h-precultivated uterine tissue samples when compared to freshly isolated controls. On the other hand, newly developing endometriotic lesions originating from the precultivated samples exhibited a much more aggressive growth throughout the 28-days observation period, as indicated by markedly increased lesion and stromal tissue volumes in our ultrasound analyses. This interesting observation may be explained by the results of our proteome profiler mouse angiogenesis array. In this array, most of the analyzed proteins were strongly upregulated in the precultivated samples, most probably due to hypoxia-induced pathways, e.g. HIF-1α/Cyr61 signaling^27,28^. Accordingly, the upregulation of pro-angiogenic factors, such as Cyr61 and KC, may have markedly increased the angiogenic activity of the tissue. Noteworthy, we also detected an upregulation of MMP-3. MMPs are proteolytic enzymes that are secreted by endometrial fragments for the breakdown and the remodeling of the extracellular matrix, which is required for the invasion into the adjacent tissue^29^. Several studies suggest that an altered expression of MMPs in eutopic and ectopic endometrium of patients may contribute to the pathogenesis of endometriosis^30,31^. Furthermore, MMP-3 has been shown to be upregulated by IL-1, which was also overexpressed in our precultivated uterine tissue samples^32^.

Additional immunohistochemical analyses of the endometriotic lesions on day 28 revealed a reduced number of MPO^+^ neutrophilic granulocytes and CD68^+^ macrophages within lesions originating from 24-h-precultivated uterine tissue samples when compared to controls. This is a surprising finding considering the fact that these immune cells have been shown to promote the growth, development, vascularization and innervation of endometriotic lesions^33,34^. Hence, based on our ultrasound results we would have expected higher immune cell numbers in lesions of 24-h-precultivated tissue samples. However, the contribution of the immune system to the pathogenesis of endometriosis is highly complex and crucially dependent on the recruitment and activation of specific immune cell subtypes^33,35^. Accordingly, we further analyzed M1 and M2 macrophage polarization within the endometriotic lesions. Of interest, we found that the number of pro-inflammatory CD86^+^ M1 macrophages was significantly reduced within endometriotic lesions originating from 24-h-precultivated uterine tissue samples when compared to controls, whereas the number of pro-angiogenic and regenerative CD163^+^ M2 macrophages did not differ between the two groups. This indicates a shift of the M1:M2 ratio towards the M2 phenotype within lesions originating from 24-h-precultivated uterine tissue samples, which may have stimulated lesion development despite low overall macrophage numbers within the ectopic endometrial tissue.

Our immunohistochemical analyses further showed that lesions developing from freshly isolated and 24-h-precultivated uterine tissue samples exhibited a comparable microvessel density on day 28. In both groups, most microvessels consisted of GFP^+^ endothelial cells, indicating their origin from the transgenic donor mice. This observation is in line with our finding that the microvessels within the uterine tissue samples survived the 24-h precultivation period. Moreover, it demonstrates that the primary vascularization mode in the present endometriosis model is inosculation, i.e. the interconnection of pre-existing microvessels inside the grafted tissue with blood vessels of the surrounding host tissue^36,37^. However, particularly in the early phase after transplantation, sprouting angiogenesis is a major prerequisite for this process^38^. Hence, the hypoxia-induced overexpression of multiple pro-angiogenic factors may have markedly accelerated inosculation within the grafted 24-h-precultivated uterine tissue samples. Accordingly, we also found an improved early vascularization of 24-h-precultivated endometrial fragments in the dorsal skinfold chamber model, as indicated by a higher functional capillary density when compared to freshly isolated controls. This improved vascularization can explain the increased growth rate of the grafts within the peritoneal cavity. Besides, it is known that hypoxia does not only induce angiogenesis, but also directly promotes cell proliferation^39^, which may have further contributed to the higher overall volume and stromal tissue volume of lesions originating from 24-h-precultivated uterine tissue samples. Finally, hypoxia stimulates the production of reactive oxygen species in endometriotic cells, which enhances the growth of endometriotic lesions^40,41^. This is mediated by the activation of different pro-inflammatory intracellular pathways, including nuclear factor-κB and cyclooxygenase-2/prostaglandin E2^41^.

In summary, the present study demonstrates that a lack of blood supply has a strong impact on the integrity, survival as well as angiogenic and proliferative activity of ectopic endometrial tissue. If such ischemic periods are too long, the tissue completely regresses and is not anymore able to develop into endometriotic lesions. In contrast, shorter ischemic periods promote the overexpression of multiple growth factors inside the tissue, resulting in the formation of endometriotic lesions of aggressive growth. Hence, the duration of the ischemic time window for shed endometrial tissue may represent a key selection factor determining the chance for the development of endometriotic lesions inside the peritoneal cavity and, thus, the risk to suffer from endometriosis.

## Materials and methods

### Animals

For this study, 12–16-week-old female C57BL/6J wild-type mice and transgenic C57BL/6-TgN(ACTB-EGFP)1Osb/J mice (Institute for Clinical and Experimental Surgery, Saarland University, Homburg/Saar, Germany) were used. The transgenic mice expressed an enhanced green fluorescent protein (EGFP) cDNA under the control of a chicken β-actin promoter and cytomegalovirus enhancer in all tissues except of hair and erythrocytes^42^. Hence, tissue transplantation from these mice into wild-type animals allowed the differentiation of pre-existing GFP^+^ microvessels originating from the tissue grafts and ingrowing GFP^−^ microvessels from the surrounding host tissue^43^.

The mice were either housed four to six (peritoneal endometriosis model) or one per cage (dorsal skinfold chamber model) on wood chips as bedding in the conventional animal facility of the Institute for Clinical and Experimental Surgery. The animals had free access to tap water and standard pellet food (Altromin, Lage, Germany) and were maintained under a 12-h day/night cycle.

All experiments were performed according to the German legislation on protection of animals and the National Institutes of Health Guide for the Care and Use of Laboratory Animals (Institute of Laboratory Animal Resources, National Research Council, Washington DC, USA) and in compliance with the ARRIVE guidelines. The study was approved by the local governmental animal protection committee (Landesamt für Verbraucherschutz, Saarbrücken, Germany; permission numbers: 21/2010, 53/2011).

### Vaginal lavage

Estrous cycles were evaluated by cytological analysis of vaginal lavage samples to exclude differences between individual animals related to different sex hormone levels. For this purpose, 15 µL of 0.9% saline solution were carefully pipetted into the vagina. The cell suspension was subsequently transferred to a glass slide and the cycle stage was determined under a phase contrast microscope (CH-2; Olympus, Hamburg, Germany). Animals in the stage of estrus were used as donor mice for the generation of uterine tissue samples and as recipient mice for the induction of peritoneal endometriotic lesions. Mice in the stage of diestrus were used for the implantation of the dorsal skinfold chamber.

### Isolation of uterine tissue samples

To generate uterine tissue samples for in vitro analyses as well as for the induction of peritoneal endometriotic lesions, C57BL/6-TgN(ACTB-EGFP)1Osb/J donor mice in the stage of estrus were anesthetized by an intraperitoneal injection of 75 mg/kg ketamine (Pharmacia GmbH, Erlangen, Germany) and 15 mg/kg xylazine (Rompun^®^; Bayer, Leverkusen, Germany). After midline laparotomy, the uterine horns of the donor mice were isolated and placed into a petri dish containing Dulbecco’s modified Eagle medium (DMEM (PAN Biotech, Aidenbach, Germany); 10% fetal calf serum, 100 U/mL penicillin, 0.1 mg/mL streptomycin; Thermo Fisher Scientific, Dreieich, Germany). The uterine horns were openend longitudinally and tissue samples with a diameter of 2 mm were carefully removed by means of a dermal biopsy punch (Stiefel Laboratorium GmbH, Offenbach am Main, Germany).

Endometrial fragments for the dorsal skinfold chamber model were also harvested from the uterine horns of anesthetized C57BL/6-TgN(ACTB-EGFP)1Osb/J mice in the stage of estrus. For this purpose, the two uterine horns were excised, placed in a petri dish containing DMEM (10% fetal calf serum, 100 U/mL penicillin, 0.1 mg/mL streptomycin; PAN Biotech, Thermo Fisher Scientific) and opened longitudinally. Subsequently, the tissue was fixed and the perimetrium was carefully removed by means of microsurgical instruments under a stereo-microscope (M651; Leica Microsystems, Wetzlar, Germany). Circular fragments with a diameter of ~ 1.3 mm were then excised from the underlying endometrium.

### Precultivation of uterine tissue samples

To simulate in vitro a variable period of retrograde menstruation, uterine tissue samples from donor mice were precultivated at 37 °C for 24 h or 72 h in 24-well petri dishes containing DMEM (10% fetal calf serum, 100 U/mL penicillin, 0.1 mg/mL streptomycin; PAN Biotech, Thermo Fisher Scientific) in a humidified atmosphere and 5% CO_2_ prior to further analyses and experiments. Freshly isolated samples (0 h) served as controls.

### Protein expression of uterine tissue samples

To analyze the expression of angiogenesis-related proteins in uterine tissue samples, a proteome profiler mouse angiogenesis array kit was used according to the manufacturer´s instruction (R&D Systems, Wiesbaden, Germany). In brief, pooled uterine tissue samples were stored in lysis buffer (10 mM Tris pH 7.5, 10 mM NaCl, 0.1 mM ethylenediaminetetraacetic acid (EDTA), 0.5% Triton-X 100, 0.02% NaN_3_, 0.2 mM phenylmethylsulphonyl fluoride (PMSF), 1:75 v/v protease inhibitor cocktail and 1:100 v/v phosphatase inhibitor cocktail (all from Sigma-Aldrich; Taufkirchen, Germany)) and homogenized. The tissue lysate was then incubated for 30 min on ice and afterwards centrifuged at 4 °C for 5 min at 16,000×*g*. The supernatants were used for whole protein extracts. A total of 250 µg protein per group was used for the array. The samples were mixed with the biotinylated detection antibody cocktail and incubated for 1 h at room temperature. Subsequently, the mixture was exposed over night at 4 °C to the capture antibodies-spotted array membrane. The visualization of the labeled specific target proteins was achieved with streptavidin–horseradish peroxidase and chemiluminescent detection reagents using an Intas ECL Chemocam Imager (Intas Science Imaging Instruments GmbH, Göttingen, Germany).

### Induction of peritoneal endometriotic lesions

To investigate the effects of precultivation on the development of peritoneal endometriotic lesions, a 24-h- and 72-h-precultivated uterine tissue sample as well as a freshly isolated control sample were transplanted into the abdominal cavity of recipient C57BL/6J mice in the stage of estrus, as previously described^44^. For this purpose, the mice were anesthetized by an intraperitoneal injection of ketamine (75 mg/kg body weight; Pharmacia) and xylazine (15 mg/kg body weight; Rompun^®^, Bayer). After midline laparotomy, the three tissue samples were randomly fixed with a 6-0 Prolene suture (Ethicon Products, Norderstedt, Germany) to the left and right abdominal wall. The laparotomy was then closed again with running 6-0 Prolene muscle and skin sutures.

### High-resolution ultrasound imaging and analysis

The development of the surgically induced endometriotic lesions was repeatedly analyzed with a Vevo 770™ high-resolution ultrasound imaging system (VisualSonics, Toronto, ON, Canada) by means of a real-time microvisualization (RMV™) 704 Scanhead (VisualSonics) with a center frequency of 40 MHz and a focal depth of 6 mm^45^. The mice were anesthetized with 2% isoflurane in oxygen, fixed in supine position on a heated stage and the abdomen was chemically depilated (Nair hair removal lotion; Church & Dwight Canada Corp., Mississauga, ON, Canada). A three-dimensional reconstruction and analysis software from VisualSonics (Vevo 770 V2.3.0) was used to analyze the ultrasound images. To measure the overall volume of developing endometriotic lesions as well as the volume of their stromal tissue and cysts (in mm^3^) by manual image segmentation, boundaries of the lesions and their cysts were outlined in parallel slices with a step size of 200 µm^46^. Moreover, we calculated the growth of lesions and stromal tissue (in % of the initial lesion and stromal tissue volume) and assessed the number of cyst-containing lesions (in %).

At the end of the in vivo experiments, the anesthetized animals were carefully laparotomized under a stereo-microscope and the largest diameter (D1) and perpendicularly aligned diameter (D2) of the endometriotic lesions were measured by means of a digital caliper. The lesion size (S) was then calculated by S = D1 * D2 * π/4^47^. Furthermore, the transplanted tissue was harvested and fixed in formalin for further histological and immunohistochemical analyses.

### Dorsal skinfold chamber model

The mouse dorsal skinfold chamber model was used to further investigate the effect of precultivation on the vascularization of developing endometriotic lesions, as previously described^48,49^.

For the implantation of the dorsal skinfold chamber, C57BL/6J mice in the stage of diestrus were anesthetized by an intraperitoneal injection of ketamine (75 mg/kg body weight; Pharmacia) and xylazine (15 mg/kg body weight; Rompun^®^, Bayer). Two titanum chamber frames (Irola Industriekomponenten, Schonach, Germany) were fixed on the dorsal skinfold of the back of the animals.The skin and muscle layers within the circular area of the observation window were removed. Thereafter, a cover glass was fixed by means of a snap ring in the observation window and the animals were allowed to recover from the anesthesia and surgical trauma for 48 h. Subsequently, the mice were anesthetized again and the cover glass of the observation window was removed. After rinsing the chamber tissue thoroughly, a 24-h- and 72-h-precultivated endometrial fragment as well as a freshly isolated control fragment were randomly placed on the striated muscle tissue within the dorsal skinfold chamber of each animal with a maximal distance to each other. Subsequently, the observation window was closed again with a new cover glass.

### Intravital fluorescence microscopy

The vascularization and size of endometrial fragments was analyzed by means of intravital fluorescence microscopy directly after transplantation (d0) into the dorsal skinfold chamber as well as on day 3, 6, 10 and 14. For this purpose, the anesthetized mice received a single intravenous injection of 0.1 mL 5% fluorescein isothiocyanate (FITC)-labeled dextran (150,000 Da; Sigma-Aldrich) into the retrobulbar venous plexus. This enhanced the imaging contrast of blood vessels by staining of intravascular blood plasma. The mice were placed on a Plexiglas stage and the vascularization of the developing endometriotic lesions was visualized by means of a Zeiss Axiotech microscope (Zeiss, Oberkochen, Germany) equipped with a 100-W mercury lamp attached to a filter block for blue, green and ultraviolet light. The microscopic images were recorded by a charge-coupled device video camera (FK6990; Pieper, Schwerte, Germany) and transferred to a DVD system for off-line evaluation. The functional capillary density, i.e. the length of red blood cell (RBC)-perfused microvessels per observation area (cm/cm^2^), and the size of the lesions (mm^2^) were assessed by means of the software package CapImage (version 8.5; Zeintl, Heidelberg, Germany). At the end of the experiment, the transplanted tissue was harvested and fixed in formalin for further histological and immunohistochemical analyses.

### Histology and immunohistochemistry

Formalin-fixed specimens of freshly isolated and precultivated uterine tissue samples as well as endometriotic lesions were embedded in paraffin. Three-µm-thick sections were cut and stained with hematoxylin and eosin (HE) according to standard procedures. In addition, sections of uterine tissue samples, which had been exposed to 0.2% trypan blue for 30 min before fixation, were counterstained with 0.1% nuclear fast red (Sigma-Aldrich). The sections were examined under a BX60 microscope (Olympus). The fraction of transplanted uterine tissue samples, which finally developed into endometriotic lesions containing endometrial stroma and glands, also referred to as take rate (%), was determined. These lesions were included in all further quantitative analyses.

For the immunohistochemical detection of proliferating and apoptotic cells in uterine tissue samples and endometriotic lesions, sections were stained with a rabbit polyclonal antibody against the proliferation marker Ki67 (1:500; Abcam, Cambridge, UK) and a rabbit polyclonal antibody against the apoptosis marker Casp-3 (1:100; New England Biolabs GmbH, Frankfurt, Deutschland). Moreover, sections of uterine tissue samples were stained with a rabbit polyclonal antibody against HIF-1α (1:50; Abcam). Additional sections of endometriotic lesions were stained with a rabbit polyclonal antibody against the lymphocyte marker CD3 (1:100; Abcam), a rabbit polyclonal antibody against the neutrophilic granulocyte marker myeloperoxidase (MPO) (1:100; Abcam), a rabbit polyclonal antibody against the pan-macrophage marker CD68 (1:100; Abcam), a rabbit polyclonal antibody against the M1 macrophage marker CD86 (1:100; New England Biolabs GmbH) and a rabbit polyclonal antibody against the M2 macrophage marker CD163 (1:200; Abcam). A goat anti-rabbit IgG biotinylated antibody (ready-to-use; Abcam) followed by avidin-peroxidase (1:50; Sigma-Aldrich) or a goat anti-rabbit peroxidase-labeled antibody (1:100; Abcam) served as secondary antibodies. 3-Amino-9-ethylcarbazole (AEC Substrate System; Abcam) was used as chromogen and counterstaining was performed with hemalaun. The fraction of proliferating, apoptotic and hypoxic cells (%) as well as the number of CD3^+^ lymphocytes, MPO^+^ neutrophilic granulocytes, CD68^+^ macrophages, CD86^+^ M1 macrophages and CD163^+^ M2 macrophages (mm^−2^) was assessed by counting the numbers of positive cells in four regions of interest within the uterine tissue samples or endometriotic lesions. Additionally, the cell density was measured by counting all cell nuclei within the stroma of uterine tissue samples (mm^−2^).

For the immunofluorescent detection of microvessels, sections were stained with a monoclonal rat anti-mouse antibody against the endothelial cell marker CD31 (1:100; Dianova GmbH, Hamburg, Germany). A goat anti-rat IgG Alexa555 antibody (1:100; Invitrogen, Darmstadt, Germany) served as secondary antibody. Cell nuclei were stained with Hoechst 33342 (2 µg/mL; Sigma-Aldrich). The microvessel density (mm^−2^) was measured using the BZ-8000 microscope (Keyence). For this purpose, the overall number of CD31^+^ microvessels was counted and divided by the area of stromal tissue.

To visualize GFP^+^ and GFP^−^ microvessels within endometriotic lesions, sections were stained with a polyclonal goat anti-GFP antibody (1:200; Biomol, Hamburg, Germany) to enhance GFP fluorescence. As secondary antibody served a biotin-labeled donkey anti-goat IgG antibody (1:100; Invitrogen) followed by Alexa488-labeled-streptavidin (1:50; Invitrogen). To detect endothelial cells, the sections were additionally stained with a monoclonal rat anti-mouse antibody against CD31 (1:100; Dianova GmbH). A goat anti-rat IgG Alexa555 antibody (1:200; Invitrogen) was used as secondary antibody. Cell nuclei were stained with Hoechst 33342 (2 µg/mL; Sigma-Aldrich). Sections solely incubated with the secondary antibodies served as negative controls. For the quantitative analysis of the fraction of CD31^+^/GFP^+^ microvessels (%) within endometriotic lesions, the sections were examined with a BX60 microscope (Olympus).

### Experimental protocol

In a first set of experiments, a total of 143 uterine tissue samples from 12 C57BL/6-TgN(ACTB-EGFP)1Osb/J mice were isolated. One third of the samples was either precultivated for 24 h or 72 h, whereas the last third served as freshly isolated control. After the cultivation period, the tissue samples were directly processed for histology, immunohistochemistry and protein expression analyses.

In a second set of experiments, a total of 66 freshly isolated as well as 24-h- and 72-h-precultivated uterine tissue samples from 4 C57BL/6-TgN(ACTB-EGFP)1Osb/J mice were transplanted into 11 C57BL/6J mice, whereby one graft of each group was randomly fixed at either the right or left abdominal wall of each recipient animal. Ultrasound image analyses of the newly developing endometriotic lesions were repeatedly performed directly after transplantation (d0) as well as on days 7, 14, 21 and 28. At the end of the in vivo experiments, the size of the lesions was assessed by means of a digital caliper. Thereafter, the lesions were excised and further processed for histology and immunohistochemistry.

In a third set of experiments, a total of 27 freshly isolated as well as 24-h- and 72-h-precultivated endometrial fragments from 4 C57BL/6-TgN(ACTB-EGFP)1Osb/J mice were transplanted into 9 dorsal skinfold chambers of C57BL/6J mice. Intravital fluorescence microscopy was performed directly after transplantation (d0) as well as on days 3, 6, 10 and 14. At the end of the experiment, the grafts were excised and further processed for histology.

### Statistical analysis

Data were first analyzed for normal distribution and equal variance. In case of parametric data, differences between two experimental groups were assessed by the unpaired Student’s t-test. In case of non-parametric data, differences between two experimental groups were assessed by the Mann–Whitney rank sum test. Differences between three experimental groups were assessed by one-way ANOVA followed by the Student–Newman–Keuls post hoc test (SigmaPlot 13.0; Jandel Corporation, San Rafael, CA, USA). All data are given as mean ± standard error of the mean (SEM). Statistical significance was accepted for P < 0.05.

## Supplementary Information

Supplementary Table S1.

## Data Availability

The datasets generated and analysed during the current study are available from the corresponding author on request.
