# Supplementary material for: The ischemic time window of ectopic endometrial tissue crucially determines its ability to develop into endometriotic lesions
Source: Sci Rep. 2022 Apr 4;12:5625. doi: 10.1038/s41598-022-09577-z (PMC8980079; doi:10.1038/s41598-022-09577-z)
Supplement: Supplementary file 1 — Supplementary Table S1. [file 41598_2022_9577_MOESM1_ESM.docx]

Supplementary Table S1: Expression of angiogenesis-related proteins in 72-h-precultivated and freshly isolated uterine tissue samples as assessed by a proteome profiler mouse angiogenesis array. Data are presented as mean pixel density ± SEM of two technical replicates and as fold change in % of freshly isolated controls.

|  | **Mean pixel density**  **(Mean ± SEM)** |  | **Fold change**  **(%)** |
| --- | --- | --- | --- |
| **Protein** | **0 h** | **72 h** | **72 h** |
| KC/CXCL1/CINC-1/GRO-alpha | 489 ± 208 | 75,593 ± 4,303 | 15,459 |
| Cyr61/CCN1, IGFBP-10 | 3,279 ± 19 | 88,894 ± 1,072 | 2,711 |
| IL-1alpha | 4,489 ± 202 | 97,641 ± 1,671 | 2,175 |
| Thrombospondin-2/TSP-2 | 8,025 ± 82 | 155,657 ± 124,424 | 1,940 |
| MCP-1/CCL2/JE | 4,931 ± 250 | 98,987 ± 209 | 1,825 |
| PIGF-2 | 4,962 ± 376 | 89,096 ± 1,797 | 1,796 |
| ADAMTS1/METH1 | 3,872 ± 137 | 63,180 ± 56 | 1,632 |
| TIMP-1 | 4,024 ± 343 | 40,782 ± 154 | 1,013 |
| KGF/FGF-7 | 823 ± 75 | 7,575 ± 310 | 920 |
| DLL4 | 1,566 ± 43 | 13,217 ± 1,011 | 683 |
| MMP-3 | 21,410 ± 30 | 126,353 ± 964 | 590 |
| DPP IV/CD26 | 7,471 ± 53 | 41,415 ± 557 | 554 |
| Amphiregulin/AR | 1,180 ± 171 | 5,717 ± 215 | 484 |
| PDGF-AA | 2,299 ± 144 | 10,464 ± 643 | 455 |
| Serpin E1/PAI-1 | 27,886 ± 521 | 126,049 ± 3,016 | 452 |
| Osteopontin/OPN | 28,734 ± 186 | 125,189 ± 254 | 436 |
| Platelet Factor 4/CXCL4/PF4 | 23,151 ± 291 | 99,136 ± 1625 | 428 |
| Endothelin-1/ET-1 | 8,289 ± 11 | 35,491 ± 230 | 428 |
| GM-CSF | 587 ± 112 | 2,282 ± 23 | 389 |
| Endoglin/CD105 | 5,148 ± 675 | 17,395 ± 423 | 338 |
| CXCL 16 | 17,205 ± 343 | 49,134 ± 1,291 | 286 |
| Fractalkine/CX3CL 1 | 3,458 ± 471 | 9,847 ± 106 | 285 |
| MIP-1alpha | 3,095 ± 109 | 7,986 ± 841 | 258 |
| PDGF-AB/BB | 3,455 ± 105 | 8,521 ± 164 | 247 |
| Pentraxin-3/PTX3/TSG-14 | 4,486 ± 194 | 11,054 ± 998 | 246 |
| HB-EGF | 2,220 ± 10 | 5,269 ± 34 | 237 |
| Angiopoietin-1/Ang-1 | 1,950 ± 209 | 4,461 ± 810 | 229 |
| IP-10/CXCL 10 | 2,303 ± 253 | 5,138 ± 7 | 223 |
| VEGF/VPF | 1,146 ± 151 | 2,532 ± 267 | 221 |
| IGFBP-3 | 59,053 ± 599 | 127,450 ± 635 | 216 |
| NOV/CCN3/IGFBP-9 | 31,010 ± 1,903 | 58,313 ± 1,822 | 188 |
| IL-10/CSIF | 2,479 ± 410 | 4,357 ± 11 | 176 |
| PD-ECGF | 1,932 ± 24 | 3,243 ± 412 | 168 |
| Angiogenin/ANG | 5,489 ± 326 | 8,510 ± 357 | 155 |
| FGF acid/FGF-1/ECGF/HBGF-1 | 26,308 ± 25 | 39,313 ± 1,040 | 149 |
| Angiopoietin-3/Ang-3 | 2,765 ± 308 | 4,116 ± 481 | 149 |
| Endostatin/Collagen VIII | 41,865 ± 1,410 | 60,856 ± 528 | 145 |
| FGF basic/FGF-2 | 13,601 ± 448 | 19,719 ± 521 | 145 |
| Proliferin | 3,814 ± 230 | 5,416 ± 436 | 142 |
| HGF | 25,854 ± 425 | 35,542 ± 289 | 137 |
| Serpin F1/PEDF | 11,456 ± 43 | 15,387 ± 326 | 134 |
| Leptin/OB | 2,983 ± 506 | 3,865 ± 225 | 130 |
| VEGF B/VRF | 4,444 ± 44 | 5,684 ± 8 | 128 |
| IGFBP-2 | 7,215 ± 142 | 9,098 ± 265 | 126 |
| Prolactin/PRL | 2,609 ± 199 | 3,234 ± 493 | 124 |
| TIMP-4 | 2,262 ± 308 | 2,396 ± 99 | 106 |
| MMP-9 | 112,331 ±21 | 117,867 ± 583 | 105 |
| SDF-1/CXCL 12 | 34,391 ± 1,770 | 35,889 ± 1,423 | 104 |
| Coagulator Factor III/Tissue Factor/TF | 54,603 ± 79 | 55,991 ± 766 | 103 |
| MMP-8 | 7,085 ± 418 | 7,201 ± 790 | 102 |
| IGFBP-1 | 3,551 ± 65 | 3,250 ± 394 | 92 |
| IL-1beta | 520 ± 77 | 414 ± 157 | 80 |
| EGF | 955 ± 28 | 618 ± 114 | 65 |
